# Draft Genome Sequences of Two Avian Pathogenic *Escherichia coli* Strains of Clinical Importance, E44 and E51

**DOI:** 10.1128/genomeA.00768-16

**Published:** 2016-08-04

**Authors:** Troels Ronco, Marc Stegger, Paal S. Andersen, Karl Pedersen, Lili Li, Ida C. N. Thøfner, Rikke H. Olsen

**Affiliations:** a National Veterinary Institute, Technical University of Denmark, Frederiksberg, Denmark; b Department of Microbiology and Infection Control, Statens Serum Institut, Copenhagen, Denmark; c College of Light Industry and Food Sciences, South China University of Technology, Guangzhou, Guangdong, People’s Republic of China; d Department of Veterinary Disease Biology, University of Copenhagen, Frederiksberg, Denmark

## Abstract

Avian pathogenic *Escherichia coli* strains have remarkable impacts on animal welfare and the production economy in the poultry industry worldwide. Here, we present the draft genomes of two isolates from chickens (E44 and E51) obtained from field outbreaks and subsequently investigated for their potential for use in autogenous vaccines for broiler breeders.

## GENOME ANNOUNCEMENT

Avian pathogenic *Escherichia coli* (APEC) causing colibacillosis in commercial poultry is an important bacterial pathogen (1). Whereas “colibacillosis” commonly refers to systemic or localized infection in broilers, ascending infections due to *E. coli* in breeders and layers may lead to infection of the reproductive tract (2), with significant impacts on animal welfare and the poultry production economy (3). Nevertheless, there are few commercially available vaccines for the protection of layers and broilers against *E. coli* infection. Consequently, the use of autogenous *E. coli* vaccines is a common practice (4). The aims of using these vaccines are two-fold: direct protection of the breeders and indirect protection of the offspring through the passage of maternally derived antibodies.

In recent years, outbreaks due to *E. coli* in broiler breeders and broilers have increased in Scandinavian countries, expediting the introduction of a new autogenous *E. coli* vaccine program for broiler breeders. Here we present the draft genomes of two *E. coli* isolates (E44 and E51) included in this autogenous vaccine.

Fragment libraries were constructed using a Nextera XT kit (Illumina) followed by 251-bp paired-end sequencing (MiSeq; Illumina) according to manufacturer’s instructions. Genomics Workbench 6.5 (CLC bio) was used for *de novo* assembly of the raw reads. It resulted in totals for size of assembly/*N*_50_ of 5,125,126 bp/83,776 bp and 5,178,940 bp/100,046 bp, total numbers of contigs of 195 and 217, and average coverages/G+C contents of 91×/50.5% and 58×/50.5% for E44 and E51, respectively.

The contigs were annotated in the NCBI Prokaryotic Genome Automatic Annotation Pipeline (PGAAP) (5). In total, E44 had 5,171 putative genes, of which 4,868 were protein-coding sequences (CDSs), whereas E51 had 5,305 putative genes, including 4,986 protein CDSs.

Various types of virulence genes that previously have been associated with APEC isolates (6) were extracted from NCBI and identified in the draft genomes using MyDbFinder 1.1 (https://cge.cbs.dtu.dk/services/MyDbFinder/). E44 carried fewer virulence genes (*fimA, fimC, iroN*, *iss, iucA, iucD, ompA,* and *vat*) than E51 (*cvaB/C*, *cvi*, *fimA*, *fimC*, *fyuA*, *ibeA*, *iroN*, *irp2*, *iss iucA*, *iucD*, and *ompA*). According to PathogenFinder (7), both E44 and E51 were predicted to be human pathogens, with probabilities of 93% and 94%, because they matched 533 and 856 pathogenic families, respectively. None of the strains carried any antibiotic resistance genes, as verified using ResFinder 2.1 (8). *In silico* typing using MLST 1.8 (9) and SerotypeFinder 1.1 (10) showed that the sequence types (STs)/serotypes of E44 and E51 were O78:H4/ST117 and O2:H5/ST140, respectively. Field production data from farms using the E44/E51-based vaccine, experimental data obtained from *in vivo* infection models, and further genome analyses could provide useful knowledge regarding development of new vaccines and insight into virulent properties.

### Accession number(s).

The two whole-genome shotgun projects have been deposited in DDBJ/ENA/GenBank under the accession numbers LXWV00000000 (E44) and LYPJ00000000 (E51). The versions described in this paper are the first versions.
